# A decade of vector control activities: Progress and limitations of Chagas disease prevention in a region of Guatemala with persistent *Triatoma dimidiata* infestation

**DOI:** 10.1371/journal.pntd.0006896

**Published:** 2018-11-06

**Authors:** Jose G. Juarez, Pamela M. Pennington, Joe P. Bryan, Robert E. Klein, Charles B. Beard, Elsa Berganza, Nidia Rizzo, Celia Cordon-Rosales

**Affiliations:** 1 Center of Health Studies, Universidad del Valle de Guatemala, Ciudad de Guatemala, Guatemala; 2 Center of Biotechnology, Universidad del Valle de Guatemala, Ciudad de Guatemala, Guatemala; 3 Centers for Disease Control and Prevention Central America Regional Office, Guatemala City, Guatemala; 4 Division of Global Health Protection, Center for Global Health, Centers for Disease Control and Prevention, Atlanta, Georgia, United States of America; 5 Visiting Investigator, Universidad del Valle de Guatemala, Ciudad de Guatemala, Guatemala; 6 Division of Vector-Borne Diseases, Centers for Disease Control and Prevention, Fort Collins, Colorado, United States of America; 7 Area de Salud de Jutiapa, Ministerio de Salud Pública y Asistencia Social de Guatemala, Guatemala; University of California Davis, UNITED STATES

## Abstract

**Introduction:**

Chagas disease, a neglected tropical disease that affects millions of Latin Americans, has been effectively controlled in Guatemala after multiple rounds of indoor residual insecticide spraying (IRS). However, a few foci remain with persistent *Triatoma dimidiata* infestation. One such area is the municipality of Comapa, Department of Jutiapa, in the southeastern region of Guatemala, where control interventions appear less effective. We carried out three cross sectional entomological and serological surveys in Comapa to evaluate a decade of vector control activities. Baseline serological (1999) and entomological (2001–2) surveys were followed by three rounds of insecticide applications (2003–2005) and intermittent focal spraying of infested houses, until approximately 2012. Household inspections to determine entomological indices and construction materials were conducted in 2001, 2007 and 2011. Seroprevalence surveys were conducted in school-age children in 1999, 2007 and 2015, and in women of child bearing age (15–44 years) only in 2015. After multiple rounds of indoor residual sprayings (IRS), the infestation index decreased significantly from 39% (2001–2) to 27% (2011). Household construction materials alone predicted <10% of infested houses. Chagas seroprevalence in Comapa declined in school-aged children by 10–fold, from 10% (1999) to 1% (2015). However, seroprevalence in women of child bearing age remains >10%.

**Conclusion:**

After a decade of vector control activities in Comapa, there is evidence of significantly reduced transmission. However, the continued risk for vector-borne and congenital transmission pose a threat to the 2022 Chagas disease elimination goal. Systematic integrated vector control and improved Chagas disease screening and treatment programs for congenital and vector-borne disease are needed to reach the elimination goal in regions with persistent vector infestation.

## Author summary

Despite the success of the Chagas disease vector control program in Guatemala, there are still regions with persistent *T*. *dimidiata* infestation. The municipality of Comapa, Jutiapa, is one of such areas. After several rounds of residual insecticide sprayings, infestation of some communities remained >15%. We evaluated the impact of a decade of vector control activities through three cross-sectional seroprevalence and entomological surveys (1999 through 2015). We found that transmission to children has decreased ten-fold. However, new infections persist in women and children. To reach the Chagas disease elimination goal, integrated vector control strategies should include community-wide insecticide applications, education to change exposure to risk factors and increased access to diagnostics and treatment for women and children.

## Introduction

Chagas disease is an important global health security threat [1,2], with over 8 million people infected, more than 10,000 annual deaths and > 528,000 disability-adjusted life years (DALYs) in Latin America alone [1,3,4]. It is caused by *Trypanosoma cruzi* and transmitted through blood transfusion, organ transplantation, congenital transmission, oral ingestion and vector-borne transmission, the most common route in endemic areas [5]. To reduce the burden of disease, control efforts have focused on the elimination of triatomine vectors from the domicile through the use of several rounds of indoor residual spraying (IRS). IRS has had great success, ranging from reducing domestic infestations to ≤1% in 17 countries, to eliminating the presence of the main vectors from entire regions [6–9]. In Central America and Mexico a community infestation of <10%, for *Triatoma dimidiata*, has been associated with a seroprevalence <1% in school-aged children (7–14 years of age) [10,11]. In Guatemala, Chagas disease control has had a broad national success in most of the endemic region, with complete elimination targeted for 2022 [6]. However, some regions have ecological, biological and social risk factors that lead to persistent infestation with *T*. *dimidiata*, a major vector species distributed from Mexico to Ecuador [12].

Guatemala was the first country in Central America to start a vector control program as part of the National Strategic Plan for Chagas Control [13]. The attack phase consisted of entomological and serological baseline surveys followed by area-wide IRS. Several rounds of IRS applications were implemented across the endemic region in the initial attack phase (2000–2003) [14]. This was followed by a surveillance phase in which community surveillance was implemented for vector infestation notification and government response [15]. It is estimated that, for the Central American region, vector-borne infections were reduced 94% by control activities during the 2000–2010 period [16]. Seropositive school-aged children decreased from 5.3% (1998) [17] to 1.3% (2005–2006) [15]. Despite the overall success, Guatemala still has some foci with *T*. *dimidiata* infestation. The municipality of Comapa, located in Southeast Guatemala, is one of such regions, where some communities have ≥15% infestation after multiple IRS interventions [12,14]. Interestingly, communities with such persistent infestation are found ≥850 meters above sea level (masl), with communities at lower altitudes remaining at <15% infestation after the initial attack phase with IRS [12].

The National Control Program continues to invest resources in control, primarily through education, house improvement programs and focalized insecticide applications in response to infestation [18]. We have analyzed entomological indices and serological data obtained during three cross-sectional studies, spanning a 15-year period in the municipality of Comapa. Our primary objective was to evaluate the effects of the Chagas control program on vector infestation and changes in human seroprevalence in school-aged children, within the context of the National Control Program activities. A secondary objective was to evaluate seroprevalence in women of child-bearing age, as a proxy to congenital transmission in the region. We discuss the implications of these findings for sustainable integrated vector control, including insecticide-based methods within a program with limited resources. Further, we discuss congenital disease prevention strategies, which together could lead to transmission interruption of *T*. *cruzi*.

## Material and methods

### Ethic statement

The serological and entomological studies conducted during the surveillance phase in 2007 received ethical approval from Universidad del Valle de Guatemala (UVG) Institutional Review Board (IRB) (Letter of approval enclosed). The serological study (2015) received ethical approval from both Universidad del Valle de Guatemala (#100-10-2014) and the Ministerio de Salud Pública y Asistencia Social (MSPAS) (01–2014) IRBs. The Centers for Disease Control and Prevention Human Subjects Research Office approved the protocol (CDC protocol #6767) with a reliance on the UVG ethics committee. We obtained individual oral (2007) and written (2015) consents from each household owner for the entomological surveys. For the serological surveys, written consents were obtained from participants. Written consent was obtained from tutor/legal guardian and assent from minors <18 years old.

### Study area and population

The data presented was collected in the municipality of Comapa, Jutiapa during three cross-sectional entomological and serological evaluations. Comapa is located at -89°54′46.8″ and 14°6′38.6748″, with an area of 132 km^2^, an average altitude of 1,200 meters and includes 56 communities. The municipality of Comapa was selected based on the persistent, high infestation indices (>15%) reported by the National Control Program of the MSPAS and Bustamante *et al* [2014]. The local ecology of Comapa is sub-tropical humid forest [19], with an average temperature of 25–29°C, relative humidity of 69–88% and an average rainfall of 200 mm during the rainy season (June-October) [20]. The population of Comapa has increased from 23,715 in 2002 [21] to an estimated 27,670 in 2013 [22]. The majority of residents live in rural conditions of poverty (72%), with more than a quarter of them living in extreme poverty [23]. The economy is based on subsistence farming and animal husbandry. Comapa has the highest illiteracy (26.5%) for a municipality in the department of Jutiapa [22].

### Timeline of studies

**Fig 1:**
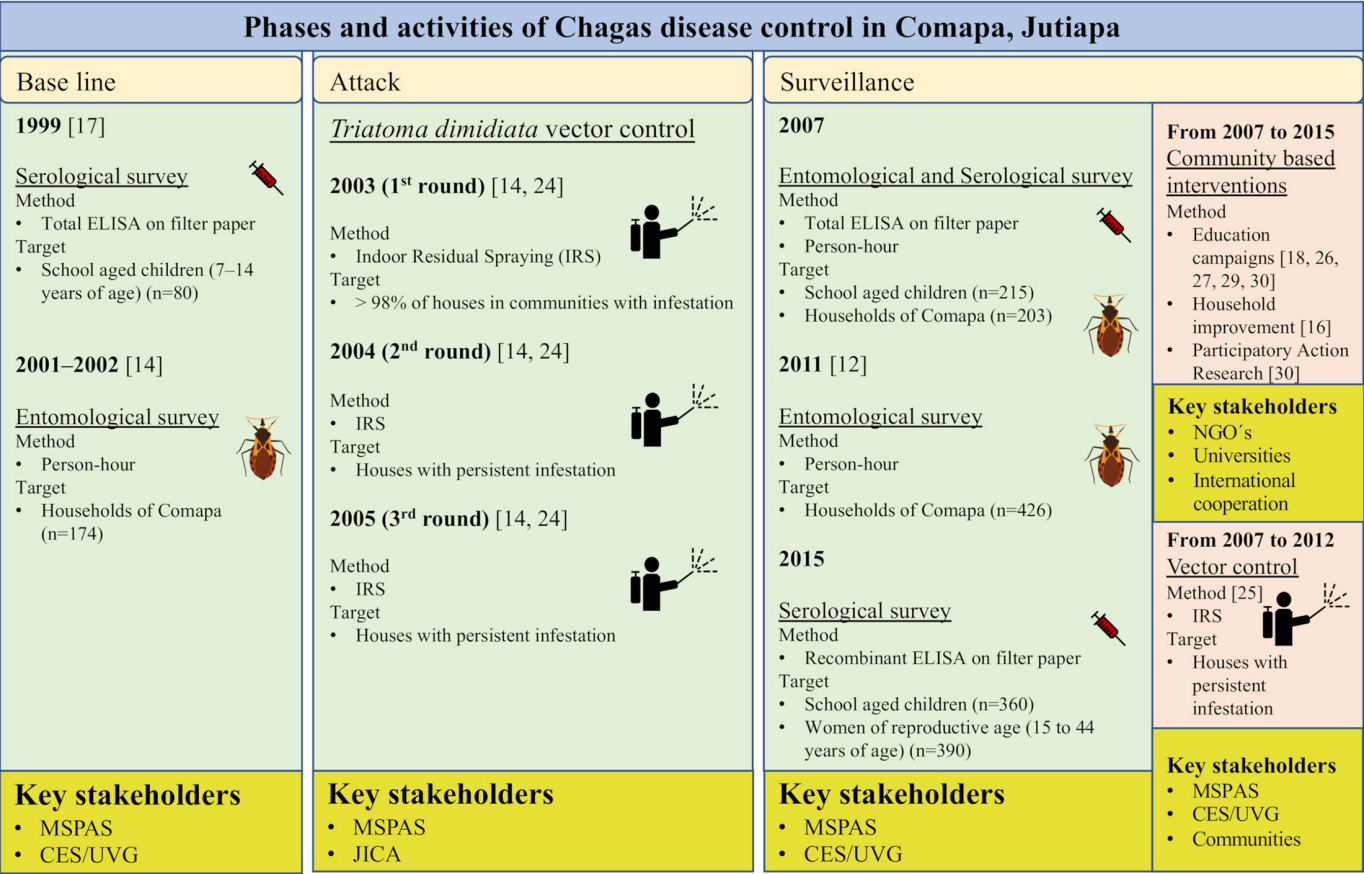
Key stakeholders and activities carried out during the last 16-years of the National Chagas Disease vector control program in Comapa, Jutiapa [12,14,30,17,18,24–29]. The green boxes show the time points of data collection and activities from 1999 till 2015. Yellow boxes show key stakeholders and pink boxes show additional vector control interventions during 2007 to 2015. MSPAS: Ministerio de Salud Publica y Asistencia Social. CES/UVG: Centro de Estudios en Salud de la Universidad del Valle de Guatemala. JICA: Japanese International Cooperative Agency.

Baseline serological [17] and entomological surveys [14] were conducted in 1999 and 2001–2002, respectively, to assess the burden of Chagas disease in the region. This was followed by three round of IRS (Attack phase), the first round in 2003 achieved >98% coverage and the 2004 and 2005 rounds targeted houses that showed persistent household infestation with *T*. *dimidiata* [14]. After this period, targeted IRS and education campaigns continued until 2012 in response to community-based surveillance [12,24–29]. Two entomological surveys were performed to evaluate vector control activities and understand risk factors. The survey in 2007 is representative of the Municipality and has not been published. The survey in 2011 focused on communities ≥850 masl and was previously published [12]. Two not yet published serological surveys were conducted in 2007 and 2015 (Surveillance phase). Vector control and evaluation activities were carried out throughout time with the support of different stakeholders, including the National Chagas Disease vector control program, Non-Governmental Organizations (NGO´s), International Cooperation and Universities (Fig 1).

### Entomological surveys (2001–2 [14], 2007, 2011 [12])

For this study we are using only the entomological results obtained from communities found ≥850 masl, which have shown to have persistent infestation with *T*. *dimidiata* over time [12]. Entomological surveys were conducted based on the person-hour method. The survey was performed by searching domicile and peridomicile environments for up to a total of one person-hour [18]. Search time could extend depending on the number of rooms, items required to be moved and/or triatomines found. All triatomines found were classified by species and developmental stage. Each specimen was stored in a 70% ethanol solution at Centro de Estudios en Salud de la Universidad del Valle de Guatemala (CES/UVG) Chagas laboratories for further analysis. At the end of the survey, the type of construction materials was recorded for wall, floor and roof top for each house.

### Community selection and sample size

#### Base line phase 2001–2 [14]

The survey was carried out by MSPAS and CES/UVG personnel between April 2001 and November 2002. Twelve communities were randomly selected within the municipality of Comapa. Houses within each community were systematically selected based on a probability proportional to size. General results from this survey where published by Hashimoto *et al*. [2006]. We used a subsample of 7 communities and 96 houses, from the original dataset.

#### Surveillance phase 2007

The survey was conducted between November and December 2007 by personnel from CES/UVG who participated in the baseline surveys. Nineteen communities were randomly selected within the municipality of Comapa. Houses with previous IRS treatment were systematically selected on a probability proportional to size, for a total of 220 houses. Sample size was based on a 20% infestation level, 2% precision, 90% CI and 80% power and a one-tailed design. We used a subsample of 17 communities and 203 houses from the original dataset.

#### Surveillance phase 2011 [12]

**Fig 2:**
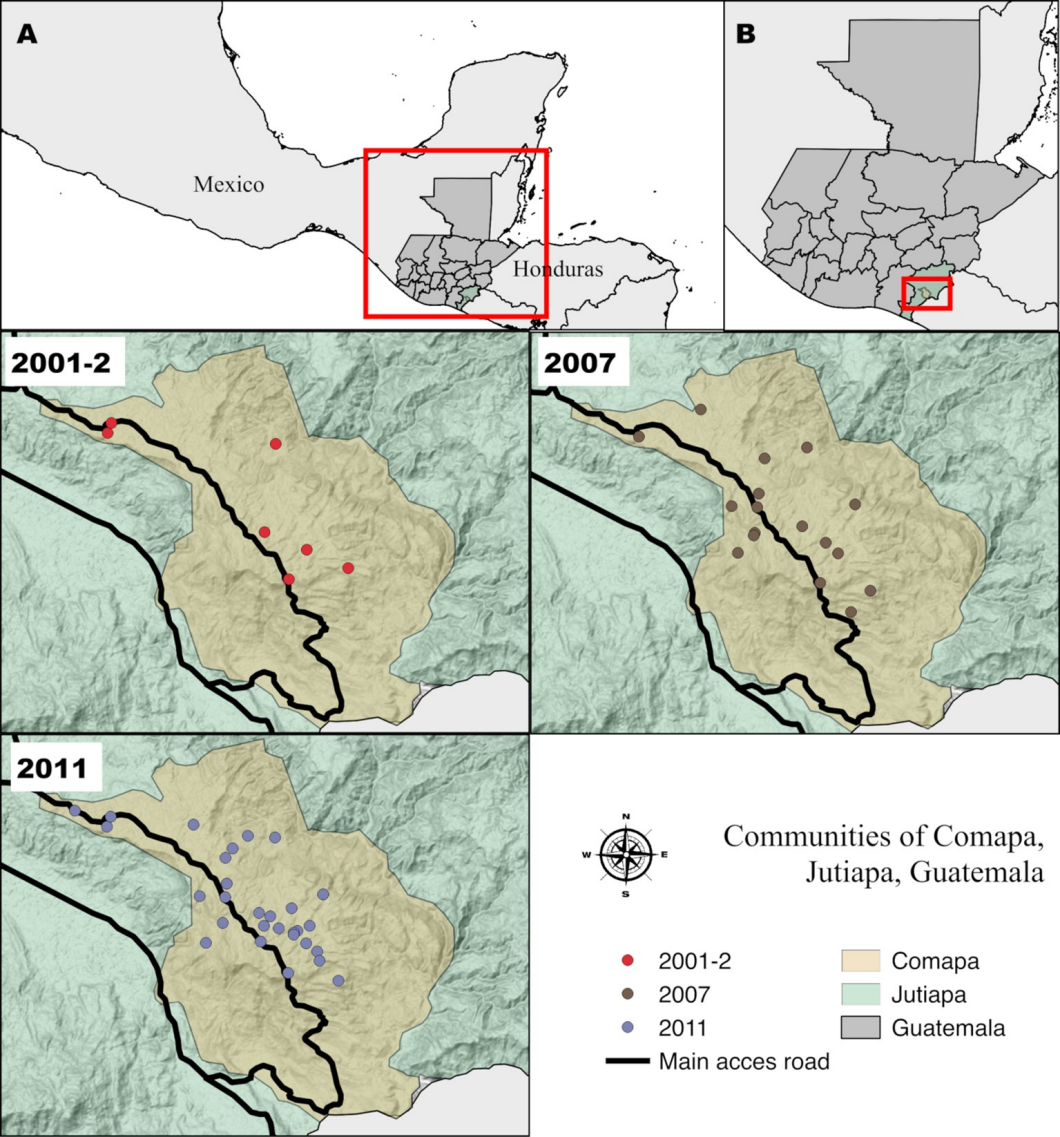
Selected communities of Comapa, Jutiapa, Guatemala, for the entomological surveys of 2001, 2007 and 2011. A) Location of Guatemala relative to Mesoamerica. B) Location of the municipality of Comapa, Jutiapa relative to Guatemala. The communities are represented by circles with red (2001), brown (2007) and gray (2011). The map was developed using QGis 2.18 with publicly available administrative boundaries.

The survey was done by MSPAS personnel and took place between January and February 2011 among 30 communities located ≥850 masl in Comapa. Houses were systematically selected, according to probability proportional to size. Results from this survey were published by Bustamante *et al*. [2014]. These communities had been surveyed and focally sprayed with deltamethrin by MSPAS personnel in 2010. The distribution of the selected communities in the three time points can be observed in Fig 2.

### Serological surveys (1999 [17], 2007, 2015)

Chagas disease serology used total antigen (1999 and 2007), as described by Rizzo *et al* [2003], or recombinant antigen (2015) ELISA tests. In each survey, serum was eluted from whole blood samples dried on filter paper. The published sensitivity and specificity were 100% and 95% for total antigen [17] and 98.8% and 99.6% for the recombinant antigen [30], respectively. While filter paper is not a recommended method of collection by the manufacturer (Wiener Lab, Argentina), the ELISA and blood spots on filter paper were chosen for the 2015 survey because this was the method used by the National Laboratory of Guatemala for surveillance procedures. We surveyed school-aged children (7–14 years of age) at three-time points during (1999, 2007, 2015) and women of child-bearing age (15–44 years) in 2015. All surveys were carried out by CES/UVG personnel.

### Individual selection and sample size

#### Base line 1999 [17]

**Fig 3:**
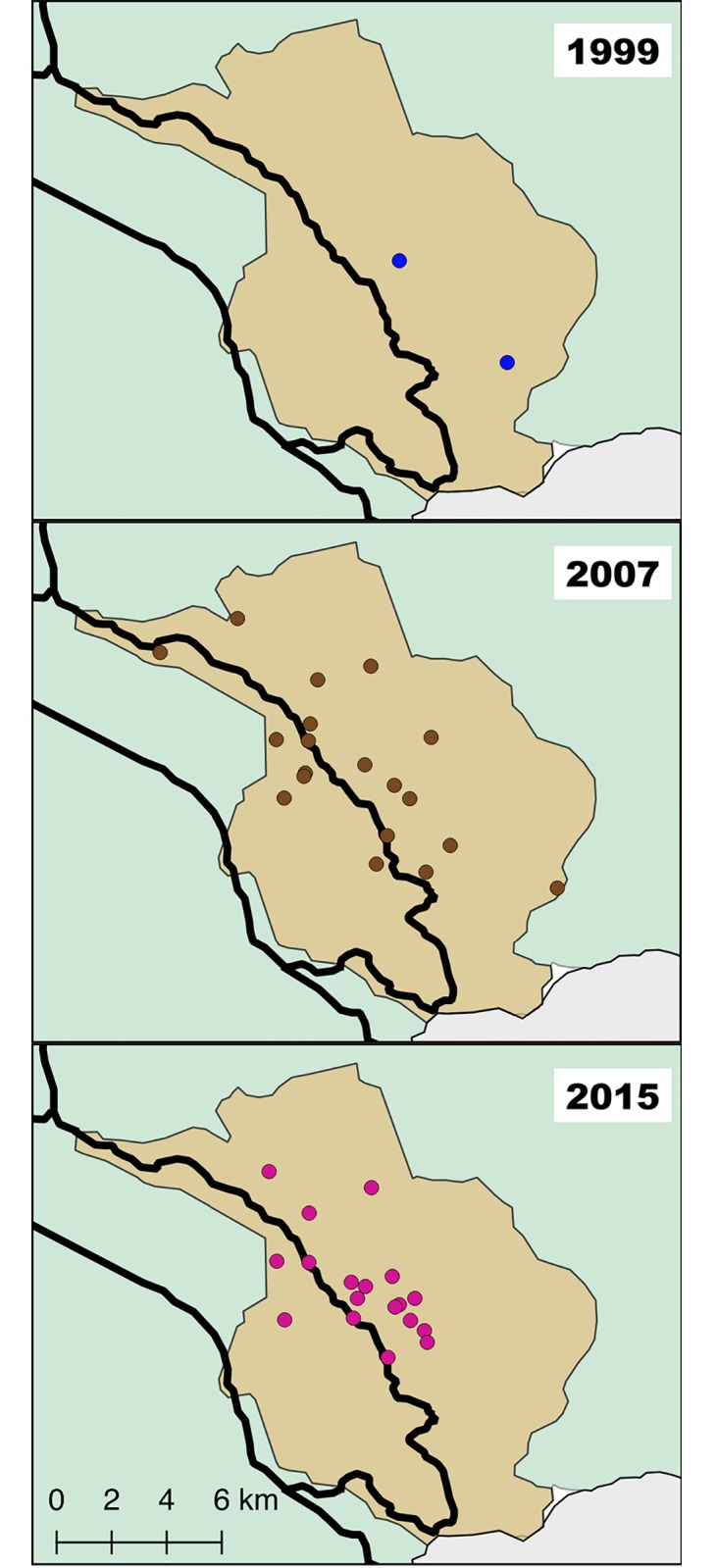
Selected communities of Comapa, Jutiapa, Guatemala, for the serological surveys of 1999, 2007 and 2015. The schools are represented by circles with blue (1999), and the communities in brown (2007) and purple (2015). The map was developed using QGis 2.18 with publicly available administrative boundaries.

The survey was carried out in October 1999 in two school districts (Fig 3) within the municipality of Comapa as part of a survey in five departments. We randomly selected 40 school-aged children within each district (n = 80). The test used was a crude ELISA. The results from this study were part of the publication by Rizzo et al. [2003]. Sampling was representative of the Municipality.

#### Surveillance phase 2007

The survey took place between November 2007 and January 2008. We randomly selected one child from each household that had been selected for the entomological survey, as described for the entomology survey of 2007 (Fig 3). The sample size was calculated using an expected seroprevalence for *T*. *cruzi* of 5%; an approximation for the targeted age group based on previous studies [17]. Statistical parameters assumed a one-tailed distribution with 95% confidence level, 90% power, and 5% precision. We analyzed 215 children from the original selection of 220 households. Sampling was representative of the Municipality.

#### Surveillance phase 2015

The survey was done between April and August 2015 in 18 communities ≥850 masl with a persistent triatomine infestation index of ≥15% in the 2011 entomological evaluation [12]. We systematically selected twenty-four households (where available) within each of the 18 communities, following the MSPAS guidelines for program evaluation (Fig 3) [18]. Sampling was designed to evaluate seroprevalence in communities considered at high risk by the MSPAS and that participated in a cluster randomized nested trial in 2012 [31]. A 20% sample size increase was added to compensate for non-compliance, for a total of 521 houses. One child and/or woman per household was randomly selected from all that lived in the house. Participants were excluded if they had received treatment for Chagas disease in the last year. A total of 360 children and 390 women were surveyed. Per MSPAS protocol, we performed serology on finger prick blood spots collected on Whatman A1 filter paper using the recombinant Chagatest ELISA v.3.0 kit (Wiener Lab, Argentina), with the following modifications. Instead of serum, we punched three 6mm blood spot circles from the filter paper and eluted at 4°C overnight in 200μl phosphate buffered saline. We combined equal volumes of eluted sample and sample diluent to obtain 100μl.

### Statistical analysis

#### Entomological surveys

Statistical analysis of *Triatoma dimidiata* entomological indices was limited to communities found ≥850 masl. We determined the relative importance (RI) of variables associated with *T*. *dimidiata* persistent domestic infestation (# of infested houses/ # of houses investigated). Houses with missing data were removed for a total sample size of 96 (2001), 203 (2007) and 422 (2011 [12]). We used a Generalized Linear Mixed Model (GLMM) with a binomial distribution and a logit link function in JMP 13.2 (SAS, USA). A pairwise correlation was performed to detect problems of multicollinearity for the predictive variables. Collinear variables were dropped from the model. The best fit model per year was evaluated with the lowest Akaike information criterion with a correction for finite samples (AICc) and Mallow’s Cp. To detect for leverage points within the dataset, we used Cook’s distance and compared the model with and without the influential observations. Additionally, odds ratio and their confidence intervals (CI) for construction variables were calculated using a Pearson χ^2^ for each survey. We are cautious with our interpretation of the variables because samples sizes were reduced to account for areas with persistent high infestation.

The infestation (# infested houses/ # surveyed houses), colonization (# infested houses with nymphs/ # surveyed houses) and density (# of triatomines/ # surveyed houses) indices were all calculated for each community based on the MSPAS guidelines [18]. To evaluate a difference for infestation, colonization and density indices over time, we carried out a Wilcoxon rank sum test and a multiple comparison with control for base line (2001) using Dunn’s method for joint ranking. Two leverage points were detected and removed from the density analysis for better interpretation of the data (S1 Dataset).

#### Serological surveys

The results for each ELISA method were corrected based on previously determined sensitivity and specificity compared with a Radio Immuno Precipitation Assay (RIPA) test [17,31]. We obtained the Blaker´s adjusted seroprevalence and Confidence Interval (CI) [32–34] for each time point and age group using Epi Tool (Ausvet) [35]. The 2007 results are interpreted with caution because the adjusted seroprevalence for 2007 included zero, being inconsistent with the assumed sensitivity and specificity of the test. Serology in women is presented in four age groups (15–22; 23–30; 31–38; 39–44), each spanning 7 years with the exception of the last group (5 years), as limited by the oldest age defined for women of childbearing age. This was performed to synchronize with the 7-year period proposed by the MSAPS for program evaluation [18] (S2 Dataset). We compared the seroprevalence using a Pearson´s Chi-square test following Campbell [36] and Richardson [37] recommendations.

### Force of infection

Force of infection was defined as the rate (number of new cases/time unit) at which susceptible individuals acquire an infectious disease [38]. To find the best force of infection model for women in high risk communities, we tested our data using two parametric (constant and Weibull) and non-parametric (cubic B-spline) models, as proposed by Samuels *et al*. [39]. The best model for our data was determined by the AIC [40] using rescaled AIC cutoffs [41] and residual plots [38]. To calculate the force of infection, the formula used was based on disease prevalence as a function of age [39]. We used the procedures suggested by Samuels *et al*. [2013] for models (catalytic and force of infection) and assumptions tested. Bootstrap 95% confidence intervals for age-specific seroprevalence and force of infection were resampled 10,000 times with replacement. During resampling, any negative incidence rates were truncated at zero [42]. Data analysis was performed in R (R Core Development Team, version 3.3.2, Vienna, Austria) using age-specific variables, since sample size was not big enough for differentiation by community (R code provided by Samuels *et al*. [2013]).

## Results

### Entomological indices and risk factors associated with *T*. *dimidiata* infestation in high risk communities

The key entomological indices obtained during the three surveys are shown in Table 1. We detected a statistically significant reduction of *T*. *dimidiata* infestation (39.2% to 26.9%), and density (1.4 to 0.6 triatomines per house) between the 2001 and 2011 surveys (Z = -2.356, df = 2, P = 0.037; Z = -4.204, df = 2, P = <0.001 respectively). No statistical difference was observed for colonization, infestation or density between 2001 and 2007 surveys. The entomological indices for the total sampled communities and houses for 2001 and 2007 are found in S1 Table.

**Table 1:** Entomological indices for communities ≥850 meters above sea level with persistent *Triatoma dimidiata* infestation in Comapa, Jutiapa.

Year	Communities	#House	Colonization	Density	Infestation
2001	7	96	28.1 (18.9–37.2)	1.42 (0.94–1.89) ^a^	39.2 (29.3–49.0) ^a^
2007	17	203	27.1 (20.9–33.2)	1.43 (0.94–1.92) ^a^	35.5 (28.9–42.1) ^a^
2011	30	422	20.5 (16.8–24.3)	0.64 (0.46–0.82) ^b^	26.9 (22.9–31.1) ^b^

^a,b^ Letters that are not shared have a statistical difference of p < 0.05. Values shown are the mean of each index (95% CI).

The predictive models were used to identify the relative importance of construction variables for *T*. *dimidiata* persistent infestation, and their ability for detecting infested households. The results showed a shift in each of the three evaluations. For the 2001 survey, bahareque (mud and stick) walls (presence), dirt floor (presence), adobe walls (presence) and cement floors (absence) showed the best fit model with an AICc of 137.810 (R^2^ = 0.092). For the 2007 survey, bahareque walls (presence), earthen floor (presence), clay roofs (absence), cinder block walls (absence) and cement floors (absence) had the best fit with an AICc of 277.381 (R^2^ = 0.066). Finally, the 2011 survey showed clay roofs (presence), adobe and bahareque walls (presence), and cement floors (absence) as the best model with an AICc of 534.824 (R^2^ = 0.06).

The odds ratio analysis showed that earthen and cement floors are the most constant housing factors associated with infestation in Comapa, but with opposite effects. Houses with earthen floors showed a higher risk for *T*. *dimidiata* infestation in all of the surveys. In contrast, houses with cement floors showed a lower risk of infestation in all of the surveys. The 2007 and 2011 surveys showed that houses with cinder block walls had a lower risk of infestation. The 2011 survey showed a higher risk of infestation for both tile roofs and bahareque walls (Table 2), as has been published before [12].

**Table 2:** Construction materials associated with *Triatoma dimidiata* infestation in high risk communities, based on the person-hour method for the 2001, 2007 and 2011 surveys.

Construction material	No. infested houses present/ houses studied Total no. (%)	Pearson χ^2^	P	OR	95% CI
2001 [14]					
Earthen floors	75/96 (78)	4.312	0.038	3.3393	1.03–10.88
Cement floors	16/96 (17)	5.497	0.019	0.1836	0.04–0.86
2007					
Cinder block walls	26/203 (13)	5.255	0.022	0.2914	0.10–0.88
Earthen floors	130/203 (64)	9.144	0.003	2.6959	1.40–5.19
Cement floors	70/203 (35)	11.169	0.001	0.3232	0.16–0.64
2011 [12]					
Tile roofs	62/422 (15)	9.974	0.002	2.4872	1.39–4.43
Bahareque walls	202/422 (48)	7.970	0.005	1.9604	1.22–3.14
Cinder block walls	108/422 (26)	8.117	0.004	0.4071	0.22–0.77
Earthen floors	285/422 (68)	18.037	<0.0001	3.6579	1.95–6.85
Cement floors	118/422 (28)	6.526	0.011	0.4706	0.26–0.85

### Seroprevalence in school-age children after IRS and multiple vector control interventions

Table 3 shows the seroprevalence for 1999, 2007 and 2015, with adjustment for test specifications. The 2007 survey had 38.1% male (82) and 61.9% (133) female participation, with a mean ± SD for age of 9.8 ± 2.2 and 10.1 ± 2.2 for male and female respectively. The 2015 survey had 49.2% (177) male and 50.8% (183) female participation with a mean of 10.6 ± 2.4 and 10.4 ± 2.3 years of age respectively. Although the 95% confidence intervals overlap for 1999 and 2007, the adjusted seroprevalence suggests a reduction in seroprevalence (Table 3). The 2007 result for the adjusted seroprevalence (<0) did not perform according to the expected specifications of sensitivity and specificity. However, we can compare this result with the baseline, assuming that the performance of the same whole-cell ELISA test is similar in different populations.

**Table 3:** Serological results from school aged children (7–14 years) tested for Chagas disease, using a crude antigen (1999 and 2007) and recombinant (2015) ELISA tests (95% CI).

Year	Population	# Children	# Positive	Apparent seroprevalence	Adjusted seroprevalence	Blaker´s exact 95% CI
1999	Municipality	80	11	13.8 (6.2–21.3)	9.2	2.6–18.8
2007	Municipality	215	9	4.2 (2.2–7.8)	<0*	0.0–2.9
2015	Persistent infestation >15%	360	7	1.9 (0.9–3.9)	1.6	0.5–3.6

1999 and 2007 = Total Antigen ELISA test sensitivity 100%, specificity 95%; 2015 = Recombinant ELISA test sensitivity 98.8%, specificity 99.6%.

*This adjusted value is not consistent with the assumed sensitivity and specificity of the test.

### Women of child-bearing age are *T*. *cruzi* seropositive

Among 390 women of childbearing age, we detected 42 (10.8%) with antibody to *T*. *cruzi*. We compared the seroprevalence of adjacent age groups for the adjusted seroprevalence in women of child bearing age (Table 4). The youngest women (15–22 years) had the lowest prevalence (1.4%) while those 39–44 years had a prevalence of 31%. We observed a statistically significant increase between age groups 23–30 (3.3%) and 31–38 (15%) (χ^2^ = 8.75; p = 0.003), also between 31–38 and 39–44 (χ^2^ = 6.07; p = 0.01). We also observed that more than 90% of all women 23–30 years old had already given birth at least once (S2 Table) and that from 1,151 children 222 were borne from seropositive mothers.

**Table 4:** Seroprevalence and force of infection in women of child bearing age (15–44 years) tested for *T*. *cruzi* antibodies in a region with persistent T. dimidiata infestation in Comapa, Jutiapa.

Age Group	Total Women	*T*. *cruzi* seropositive	Apparent seroprevalence (95% CI)	Adjusted seroprevalence	Blaker´s exact 95% CI	Force of infection per year
**15–22**	114 (29.2)	2	1.8 (0.5–6.2)	1.4 ^a^	0.0–5.6	0.2%
**23–30**	109 (27.9)	4	3.7 (1.4–9.1)	3.3 ^a^	0.9–8.6	0.9%
**31–38**	99 (25.4)	15	15.2 (9.4–23.5)	15 ^a^^,^^b^	7.8–23.5	2%
**39–44**	68 (17.4)	21	30.9 (21.2–42.6)	31^b^	20.6–43.1	3.6%
**Total**	390 (100)	42	10.8 (8.1–14.2)	10.5	7.7–13.7	1.6%

^a,b^ Letters that are not shared have a statistical difference of p < 0.05.

**Fig 4:**
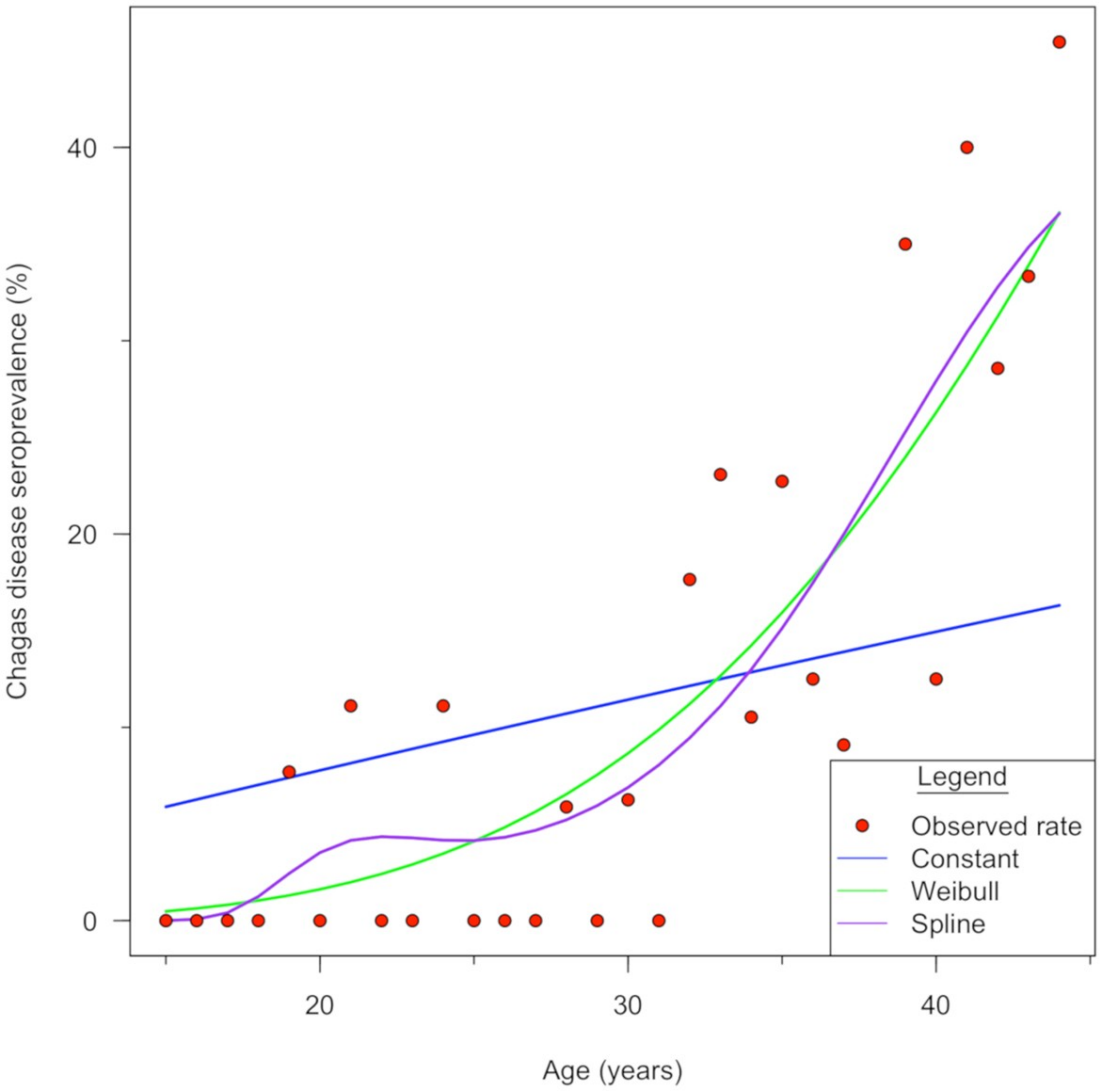
Risk of seroprevalence increases with age. The Weibull catalytic model shows the best fit of estimated seroprevalence to calculate the force of infection. Observed seroprevalence and estimated seroprevalence of the three catalytic models. The red dots represent the observed rate: the blue, green and purple lines represent the Constant, Weibull and Spline catalytic models respectively.

The Weibull catalytic model showed the best fit for our data with the lowest AIC = 228 in comparison to the Constant model (AIC = 248, X^2^ = 22.04, df = 1, p< 0.0001) and B spline (AIC = 233, X^2^ = 1.49, df = 3, p = 0.6), even though no statistical difference was detected for the latter (Fig 4). The Weibull model showed that the force of infection has an exponential increase over time in communities with persistent infestation. We used the Weibull catalytic model, as an estimation of incidence. The mean force of infection in women 15–44 years of age was 1.6% per year. We observed that the force of infection remained < 1% per year up to 27 years of age (13-year period). Force of infection reached 2% per year at 35 (8-year period), 3% per year at 40 (5-year period) and 4% per year at 43 (3-year period) years of age.

## Discussion

The Chagas disease vector control program has successfully controlled *T*. *dimidiata* domestic infestation in most of the department of Jutiapa [24]. However, areas such as Comapa show evidence of persistent infestation. In this region, infestation with *T*. *dimidiata* decreases immediately after IRS but recovers to the original levels as the insecticides decay [10,12]. To evaluate the impact of the vector control program in these areas, three cross-sectional entomological surveys were conducted and showed significant reductions in infestation and density. The presence of cement floors was consistently a protective factor for infestation. Serological surveys of school-aged children over a period of 15 years, show reduced transmission after 10 years of vector control activities. Finally, because of new opportunities to detect and treat congenital infections, we performed a cross-sectional serological survey of four age groups among women of child-bearing age in 2015 (15–44 years of age) living in communities with persistent infestation. The information presented is intended to guide public health officials in evaluation and possible revision of activities and goals for transmission elimination in such regions [18].

We observed a significant decrease of infestation and density indices from baseline to the surveillance phase in 2011. However, these communities had been recently intervened with focalized spraying a few months prior to our final evaluation. Thus, we interpret these results with caution, since these lower indices could be a consequence of recent sprayings, as seen by Hashimoto *et al* [2006]. We observed that IRS alone did not decrease vector infestation <15% and these communities are still considered at high risk for vector-borne transmission [18]. In Comapa, *Triatoma dimidiata* shows a well domesticated cycle with infestation and colonization indices all year long [43]. Interestingly, *Triatoma dimidiata* has a much lower density index than other triatomine vectors such as *R*. *prolixus* [44] and *T*. *infestans* [45]. We believe that high infestation/colonization indices with low domestic densities are due to non-domiciliary populations that move between the domicile and peridomicile [12] a seasonal behavior also observed in Yucatan [43]. We suggest that integrated interventions, including widespread education, improved spraying strategies for domicile and peridomicile, removal of animals from houses, and improved housing are needed for sustainable vector control in this region [31,46]. We do not believe wide spraying campaigns, as the ones carried out during the initial attack phase, would be adequate or sustainable for regions with persistent infestation. This is an unsustainable method of control financially and studies done in Honduras have shown that the second round of IRS is not as effective [47], something we also observed when comparing the baseline (2001–2) to the first surveillance phase (2007). Current vector control guides indicate only focalized spraying when the community reports infestation [18]. We propose that community-wide sprayings should be performed in communities with >15% infestation.

In the region of Jutiapa, construction materials have been shown to provide either a protective or a risk factor in *T*. *dimidiata* domestic infestation [12,25]. These risk factors are sometimes used to guide the selection of houses to be surveyed or intervened within a community by field personnel. However, our predictive model showed that construction materials alone should not be used as a method of selection, since they can predict <10% of infested houses. Nonetheless, risk factors associated with construction materials should be addressed and evaluated in vector control activities. During the three cross-sectional entomological surveys, we consistently detected that earthen and cement floors showed a higher and lower risk, respectively, for *T*. *dimidiata* infestation. These factors have also been associated with rodent reservoirs of *T*. *cruzi*, which were targeted for a community-based intervention to reduce transmission risk [12,31]. We also observed that other construction material, such as cinder block walls, consistently showed a lower risk for the 2007 and 2011 surveys. We believe that education regarding other risk factors, such as the presence of dogs, chickens and rodents in the household, should be included in vector control campaigns [12].

Results from the serological surveys in school-aged children performed in 2015 in communities with >15% infestation suggest that vector-borne transmission has decreased, ten-fold. However, infection persists at a low level in the region. Adjusted results from 1999 and 2015 should be compared with caution, because the populations sampled are different. However, the prevalence in high risk communities at baseline would be expected to be at least as high as that observed at the municipality level and there is an overall reduction. We need to note that these serological results are similar as those seen by Hashimoto *et al* [2012] in regions where multiple rounds of IRS were used for the elimination of *Rhodnius prolixus*. We also observed that women in the 15–30 year of range, who were <15 years old when vector control activities started in the region, have maintained a force of infection <1% per year. It should be noted that women of 15–22 years old, who were less than nine years old before the first IRS, show similar seroprevalence (1.4%) compared with school-aged children in 2015 (1.6%). This suggests that there has been no apparent increase in infection over time. Insecticide applications have been shown to temporarily reduce the force of infection in Bolivia as well [39].

A limitation of the study is the lack of systematic data on previous spraying activities at the household level, the seasonality for each of the entomological surveys and the personnel conducting the person-hour method. The serological studies have two limitations. Although the 1999 and 2007 surveys were designed to represent the Municipality and used the same whole antigen ELISA test, the 2007 survey sample size was calculated for a one-tailed distribution. The aberrant results obtained after adjusting for test specifications suggest that the sample size might be insufficient and the adjusted results from 2007 should be interpreted with caution. In addition, the 2015 serological sampling was not designed for comparison with the previous surveys, representing only the communities at highest risk. However, we are being conservative with our analysis assuming that this communities at high risk had at least the same level of seroprevalence as for the municipality.

We show that communities with persistent infestation benefit from focalized IRS. This had temporary effects on infestation and density, decreasing transmission risk, as observed by Gürtler *et al*. [2007] in el Chaco, Argentina for *T*. *infestans*. The 10.5% seroprevalence in women of child-bearing age suggests that prenatal Chagas screening should be implemented in this region. In a previous study we reported a 3.5% seroprevalence at the Comapa health center [48]. Improving access to diagnostics at the local level should be a priority to reach women living in communities at highest risk [48]. Serological studies on this group should be conducted in areas where control of triatomine infestation was achieved, to include Chagas as a prenatal test as part of the framework for the elimination of mother-to-child transmission of HIV, Syphilis, Hepatitis B, and Chagas [49]. Infants with congenital *T*. *cruzi* benefit from benznidazole treatment since it has shown to be well-tolerated and very effective in treating congenital infections [2], now the most common type of infection in some countries. Treatment of congenital infection with a new formulation simplifies the treatment of newborns with the correct dosage per tablet (https://www.dndi.org/achievements/paediatric-benznidazole/).

Integrated vector management strategies are needed for more sustainable effects [47]. Control efforts should be maintained and optimized, since persistent infestation levels pose a constant threat for increased densities and renewed transmission. Defunding vector control activities can lead to renewed transmission [50,51], and loss of the current success achieved by the program. Even though focalized IRS did not sustain infestation reduction below 5% [18], it did have an impact in transmission reduction. Thus, new strategies should include IRS as a public health measure in areas with persistent infestation. Maintaining the combined control efforts of IRS, community-based educational and housing interventions, together with screening of pregnant women and treatment of congenital and acute infections will be required to reach the 2022 Chagas disease elimination goal [6].

## Supporting information

S1 Checklist STROBE checklist. Checklist of items that should be included in reports of ***cross-sectional studies*.**
(DOC)

S1 Dataset Entomological surveillance of *Triatoma dimidiata* in Comapa, Jutiapa. The dataset contains the information of the 2001, 2007 and 2011 entomological surveys conducted in collaboration by CES and the MSPAS.
(XLSX)

S2 Dataset Serological evaluations of school aged children (1999, 2007 and 2015) and women between 15–44 years of age (2015) for Chagas disease. The dataset shows the results obtained from different serological evaluations conducted in the municipality of Comapa, Jutiapa.
(XLSX)

S1 Table *Triatoma dimidiata* entomological indices for Comapa, Jutiapa. Values shown are the mean of each index (SE).
(DOCX)

S2 Table Reproductive information of women 15–44 years of age tested for Chagas disease seroprevalence in the municipality of Comapa, Jutiapa. (DOCX)
