# Supplementary material for: A decade of vector control activities: Progress and limitations of Chagas disease prevention in a region of Guatemala with persistent Triatoma dimidiata infestation
Source: PLoS Negl Trop Dis. 2018 Nov 6;12(11):e0006896. doi: 10.1371/journal.pntd.0006896 (PMC6239342; doi:10.1371/journal.pntd.0006896)
Supplement: S1 Table — Values shown are the mean of each index (SE). (DOCX) [file pntd.0006896.s004.docx]

**S1 Table. *Triatoma dimidiata* entomological indices for Comapa, Jutiapa. Values shown are the mean of each index (SE).**

| Year | Communities | #House | Colonization index | 95% CI | Density index | 95% CI | Infestation index | 95% CI | Dispersion index |
| --- | --- | --- | --- | --- | --- | --- | --- | --- | --- |
| 2001 | 12 | 174 | 21.2 (5.9) | 8.3–34.1 | 1.60 ^a^ (0.68) | 0.11–3.09 | 29.6 (8.4) | 11.0–48.1 | 75.0 |
| 2007 | 19 | 220 | 22.6 (4.8) | 12.5–32.8 | 1.29 ^a^ (0.30) | 0.63–1.93 | 29.5 (6.0) | 16.9–42.1 | 89.5 |

^a,b^ Letters that are not shared have a statistical difference of p < 0.05.
