# Supplementary material for: A decade of vector control activities: Progress and limitations of Chagas disease prevention in a region of Guatemala with persistent Triatoma dimidiata infestation
Source: PLoS Negl Trop Dis. 2018 Nov 6;12(11):e0006896. doi: 10.1371/journal.pntd.0006896 (PMC6239342; doi:10.1371/journal.pntd.0006896)
Supplement: S2 Table — (DOCX) [file pntd.0006896.s005.docx]

**S2 Table. Reproductive information of women 15-44 years of age tested for Chagas disease seroprevalence in the municipality of Comapa, Jutiapa.**

| Age Group | Total Women (%) | Women with children (%) | ≤ 4 children  (%) | ≥ 5 children  (%) |
| --- | --- | --- | --- | --- |
| 15-22 | 114 (29.2) | 31 (27.2) | 31(100) | 0 (0) |
| 23-30 | 109 (27.9) | 103 (94.5) | 95 (92.2) | 8 (7.8) |
| 31-38 | 99 (25.4) | 97 (97.8) | 55 (56.7) | 42 (43.3) |
| 39-44 | 68 (17.4) | 62 (91.2) | 18 (29.0) | 47 (75.8) |
| Total | 390 (100) | 296 (75.9) | 199 (67.2) | 97 (32.8) |
